# Stillbirths in China: a nationwide survey

**DOI:** 10.1111/1471-0528.16458

**Published:** 2020-09-02

**Authors:** J Zhu, J Zhang, H Xia, J Ge, X Ye, B Guo, M Liu, L Dai, L Zhang, L Chen, Y Wang, X Wang, H Liu, C Chen, Y Wang, G Wang, M Cai, X Yang, F Li, C Fan, Y Ruan, L Yu, R Zhang, H Xu, J Zhang, X Ma, D Yuan, Y Zhu, D Wang, AP Betran, H Qi, T Duan, J Zhang, for the China Labor and Delivery Survey

**Affiliations:** ^1^ MOE‐Shanghai Key Laboratory of Children's Environmental Health Xinhua Hospital Shanghai Jiao Tong University School of Medicine Shanghai China; ^2^ Department of Obstetrics and Gynecology Xinhua Hospital Shanghai Jiao Tong University School of Medicine Shanghai China; ^3^ Department of Obstetrics The Maternal and Child Health Hospital of Guangxi Zhuang Autonomous Region Nanning China; ^4^ Department of Obstetrics Shijiazhuang Obstetrics and Gynecology Hospital Hebei China; ^5^ Department of Obstetrics and Gynecology Nanjing Drum Tower Hospital Nanjing University Medical School Jiangsu China; ^6^ Department of Obstetrics The Maternal and Child Health Hospital of Dongchangfu District Shangdong China; ^7^ Department of Obstetrics Shanghai First Maternity and Infant Hospital Tongji University School of Medicine Shanghai China; ^8^ Department of Obstetrics The Maternal and Child Healthcare Hospital of Xiangtan Hunan China; ^9^ Department of Obstetrics Qihetai Maternal and Child Health Hospital Heilongjiang China; ^10^ Department of Obstetrics Fujian Provincial Maternity and Children's Hospital Fujian China; ^11^ Department of Obstetrics and Gynecology Suzhou Municipal Hospital Nanjing University Medical School Jiangsu China; ^12^ Department of Obstetrics and Gynecology Traditional Chinese Medicine Integrated Hospital of Tongzhou District Beijing China; ^13^ Department of Obstetrics Northwest Women's and Children's Hospital Xi'an Jiao Tong University Shaanxi China; ^14^ Department of Obstetrics Maternity and Child Care Center of Xingyang Henan China; ^15^ Department of Obstetrics and Gynecology Wenzhou People's Hospital Wenzhou Maternal and Child Health Care Hospital The Third Clinical Institute Affiliated to Wenzhou Medical University Wenzhou Zhejiang China; ^16^ Department of Obstetrics Inner Mongolia Maternity and Child Health Care Hospital Hohhot Inner Mongolia Autonomous Region China; ^17^ Department of Obstetrics Changsha Hospital for Maternal and Child Health Care Hunan China; ^18^ Department of Obstetrics and Gynecology The First Affiliated Hospital of Chongqing Medical University Chongqing China; ^19^ Department of Obstetrics Haidian Maternal and Child Health Hospital Beijing China; ^20^ Department of Obstetrics and Gynecology Renmin Hospital of Wuhan University Hubei China; ^21^ Department of Obstetrics Beijing Obstetrics and Gynecology Hospital Capital Medical University Beijing China; ^22^ Department of Obstetrics and Gynecology Jinhua People's Hospital Zhejiang China; ^23^ Department of Obstetrics and Gynecology Wenling Maternal and Child Health Hospital Zhejiang China; ^24^ Department of Obstetrics Shaoxing Maternal and Child Health Hospital Zhejiang China; ^25^ Department of Obstetrics and Gynecology The Fifth Hospital of Xiamen Fujian China; ^26^ Department of Obstetrics Gansu Provincial Maternity and Child‐care Hospital Lanzhou Gansu China; ^27^ Department of Obstetrics Gaizhou Maternal and Child Health Hospital Liaoning China; ^28^ Department of Obstetrics and Gynecology Jiangyin People's Hospital Southeast University School of Medicine Jiangsu China; ^29^ Department of Obstetrics and Gynecology The First Hospital Affiliated to Army Medical University Chongqing China; ^30^ UNDP/UNFPA/UNICEF/WHO/World Bank Special Programme of Research, Development and Research Training in Human Reproduction Department of Reproductive Health and Research World Health Organization (WHO) Geneva Switzerland

**Keywords:** Antepartum, epidemiology, intrapartum, stillbirth

## Abstract

**Objective:**

To estimate a stillbirth rate at 24 or more gestational weeks in 2015–2016 and to explore potentially preventable causes in China.

**Design:**

A multi‐centre cross‐sectional study.

**Setting:**

Ninety‐six hospitals distributed in 24 (of 34) provinces in China.

**Population:**

A total of 75 132 births at 24 completed weeks of gestation or more.

**Methods:**

COX Proportional Hazard Models were performed to examine risk factors for antepartum and intrapartum stillbirths. Population attributable risk percentage was calculated for major risk factors. Correspondence analysis was used to explore region‐specific risk factors for stillbirths.

**Main outcome measures:**

Stillbirth rate and risk factors for stillbirth.

**Results:**

A total of 75 132 births including 949 stillbirths were used for the final analysis, giving a weighted stillbirth rate of 13.2 per 1000 births (95% CI 7.9–18.5). Small for gestational age (SGA) and pre‐eclampsia/eclampsia increased antepartum stillbirths by 26.2% and 11.7%, respectively. Fetal anomalies increased antepartum and intrapartum stillbirths by 17.9% and 7.4%, respectively. Overall, 31.4% of all stillbirths were potentially preventable. Advanced maternal age, pre‐pregnant obesity, chronic hypertension and diabetes mellitus were important risk factors in East China; low education and SGA were major risk factors in Northwest, Southwest, Northeast and South China; and pre‐eclampsia/eclampsia and intrapartum complications were significant risk factors in Central China.

**Conclusions:**

The prevalence of stillbirth was 13.2 per 1000 births in China in 2015–2016. Nearly one‐third of all stillbirths may be preventable. Strategies based on regional characteristics should be considered to reduce further the burden of stillbirths in China.

**Tweetable abstract:**

The stillbirth rate was 13.2 per 1000 births in China in 2015–2016 and nearly one‐third of all stillbirths may be preventable.

## Tweetable abstract

The stillbirth rate was 13.2 per 1000 births in China in 2015–2016 and nearly one‐third of all stillbirths may be preventable.

## Introduction

Approximately 2.6 million stillbirths in late pregnancy occur globally every year.^1^, ^2^ Compared with the accelerating progress in reducing maternal mortality and mortality in children under 5 years of age, the progress in reducing worldwide stillbirths remains slow and insufficient. Despite the devastating effects, particularly on parental mental health, and large direct and indirect costs,^3^ stillbirths have been neglected in public health debates for a long time. They are often not included in national and international records and registrations despite stillbirth rates indicating the quality of perinatal care.^4^ Recently, attention has been drawn to this long‐standing issue. The Every Newborn Action Plan by the World Health Organization (WHO) in 2014 set a target of 12 or fewer stillbirths per 1000 births in all countries by 2030.^5^

Based on a modelling estimate, the stillbirth rate in China has declined by half since 2000, from 14.5 per 1000 births in 2000 to 7.2 per 1000 births in 2015. But China accounts for approximately one‐fifth of world’s population. The number of stillbirths still ranked the fourth highest in the world (122 000 stillbirths per year).^1^ In 2016, using data from China’s National Maternal Near Miss Surveillance System, Zhu et al.^6^ reported a third trimester stillbirth rate of 8.8 per 1000 births between 2012 and 2014. To reduce the burden of stillbirth further, data monitoring and understanding of the causes for stillbirth are essential to create and implement effective interventions. Furthermore, the regional variation in China is large and the preventive strategy must be site‐specific. Unfortunately, national estimates on stillbirth rates are still limited and in‐depth epidemiological analyses missing.

In addition, as advances in neonatal intensive care have raised fetal viability at 24–28 gestational weeks in high‐income countries and certain areas in China,^7^, ^8^ suggestions have been made to count fetal deaths before 28 weeks of gestation. It is also crucial to improve our understanding of the causes and factors associated with stillbirths. For example, antepartum and intrapartum fetal deaths differ substantially in various characteristics. Data from the USA have further suggested that one‐quarter of stillbirths were potentially preventable,^9^ but whether this estimate is transferable to China remains unclear.

This study used data from the China Labor and Delivery Survey to estimate a stillbirth rate from 24 weeks of gestation, to describe the causes and factors associated with antepartum and intrapartum stillbirths, separately, and to explore potentially preventable causes in China.

## Methods

### Study design and sample

The China Labor and Delivery Survey was a multi‐centre cross‐sectional study throughout the country conducted between 1 March 2015 and 31 December 2016. Participating hospitals were approached through obstetric networks. Hospitals with 1000 or more deliveries per year were eligible. Depending on the annual delivery volume, 5–10 consecutive weeks were randomly selected in a 12‐month period as the study window. Within the selected weeks, all births at 24 completed weeks of gestation or more or a birthweight of ≥500 g were included. Medical records were retrieved and de‐identified information on maternal socio‐demographic characteristics, medical and pregnancy histories, pregnancy and labour complications and perinatal outcomes was extracted by trained staff. A data extraction protocol and a manual of operation were developed to guide data extraction. The staff, mostly nurses and midwives at the labour and delivery unit, were trained by senior professional staff. The completed data extraction forms were reviewed by the data manager for completeness before they were entered into the database. The data management system was programmed with built‐in logic checks to validate the consistency of the related variables and plausible values. A detailed description on sampling and data management was published elsewhere.^10^

A total of 96 hospitals distributed in 24 (of 34) provinces, autonomous regions and municipalities in China were included in the analysis. This study was approved by the Ethics Review Board of the Xinhua Hospital Affiliated to the Shanghai Jiao Tong University School of Medicine (XHEC–C–2015–006), the WHO Research Ethics Review Committee (HRP Study A65899) and participating hospitals.

### Definitions

Stillbirth was defined as a baby born with no signs of life at a gestational age of 24 weeks or more. Gestational age was ascertained on the basis of the last menstrual period or of ultrasound dating in the first trimester when the date of the last menstrual period was uncertain. An intrapartum stillbirth was defined as a fetal death occurring after the onset of labour but before birth, and an antepartum stillbirth as a fetal death occurring before the onset of labour. We used the standard partition for geographical regions in China (East, North, South, Central, Northeast, Northwest and Southwest).^11^ Hospital levels were determined officially by local governments.^12^

Maternal pre‐pregnancy body mass index (BMI) was categorised as follows: underweight <18.5 kg/m^2^, normal 18.5–23.9 kg/m^2^, overweight 24–27.9 kg/m^2^ and obese ≥28 kg/m^2^.^13^ Severe small for gestational age (SGA) was based on a birthweight below the 3rd percentile for a given gestational week, using a global reference for fetal weight and birthweight percentiles.^14^ Post‐term pregnancy was defined as pregnancy lasting longer than 42 gestational weeks. Sexually transmitted diseases included HIV, syphilis, gonorrhoea and chlamydia trachomatis. Intrapartum complications included prolapse of the cord, fetal heart rate abnormality, shoulder dystocia, severe birth trauma and prolonged labour. Placenta praevia and placenta abruption were considered complications of the placenta.

We further categorised causes of stillbirths into potentially preventable causes, fetal causes and other causes. Fetuses with major structural or genetic anomalies were classified as fetal causes. We developed the potentially preventable causes based on a previously published definition^9^ and current obstetric knowledge and practices, which included maternal medical conditions, gestational hypertension, pre‐eclampsia/eclampsia, gestational diabetes, SGA, preterm premature rupture of the membranes (PPROM), intrapartum complications, post‐term pregnancy and sexually transmitted diseases. The remaining causes, such as placenta praevia, placenta abruption, multiple pregnancy and other unknown causes were classified as other causes.

### Statistical analysis

The 2016 China Statistical Yearbook provided the number of deliveries in each province.^11^ The annual number of births in each province was stratified by hospital levels. We assigned each birth a weight based on the inverse probability weighting, taking into account the number of births in the province with the same hospital level and the number of records reviewed in the hospital with the same hospital level.^10^

We performed time‐to‐event analysis using COX Proportional Hazard Models to assess the association between maternal characteristics and stillbirths, compared with live births, by taking into account of the sampling strategy and clustering of births within hospitals, using the PROC SURVEYPHREG procedure in SAS (Cary, NC, USA).^15^ The proportional hazard assumption was tested using Shoenfeld residuals, which were plotted against each covariate and the graphs inspected for any trend in the residuals. We reported the crude hazard ratio (HRs) with 95% confidence interval (CI) and adjusted HR controlling for maternal age, race, insurance, education, parity, pre‐pregnant BMI, previous pregnancy loss, previous stillbirth, previous preterm birth, hospital location and hospital levels. We further examined the association between medical complications and stillbirths by the same approach.

To assess the proportion of stillbirths that could be potentially prevented if risk factors were removed, the population attributable risk percentage (PAR%)^16^ was calculated for each important risk factor. The PAR% was interpreted in this study as the percent incidence of stillbirth in the population that would be eliminated if the health condition (e.g. pre‐eclampsia) were eliminated.

The overall region‐specific and cause‐specific stillbirth rates were calculated by the PROC SURVEYFREQ procedure in SAS, taking into account of the sampling strategy and clustering of births within hospitals. Stillbirths per 1000 births and stillbirths per 1000 fetuses at risk by week of gestation were used to calculate the stillbirth rate.^17^, ^18^ The weighted proportion of categorised causes of stillbirths, i.e. potentially preventable, fetal and other causes in each geographical region was presented as well. We further performed the Correspondence Analysis among stillbirths to explore the relationship between risk factors and geographical regions. SAS version 9.4 was used for all statistical analyses.

### Patient and public involvement

Patients and the public were not involved in the design, conduct or reporting in our study.

## Results

**Figure 1:**
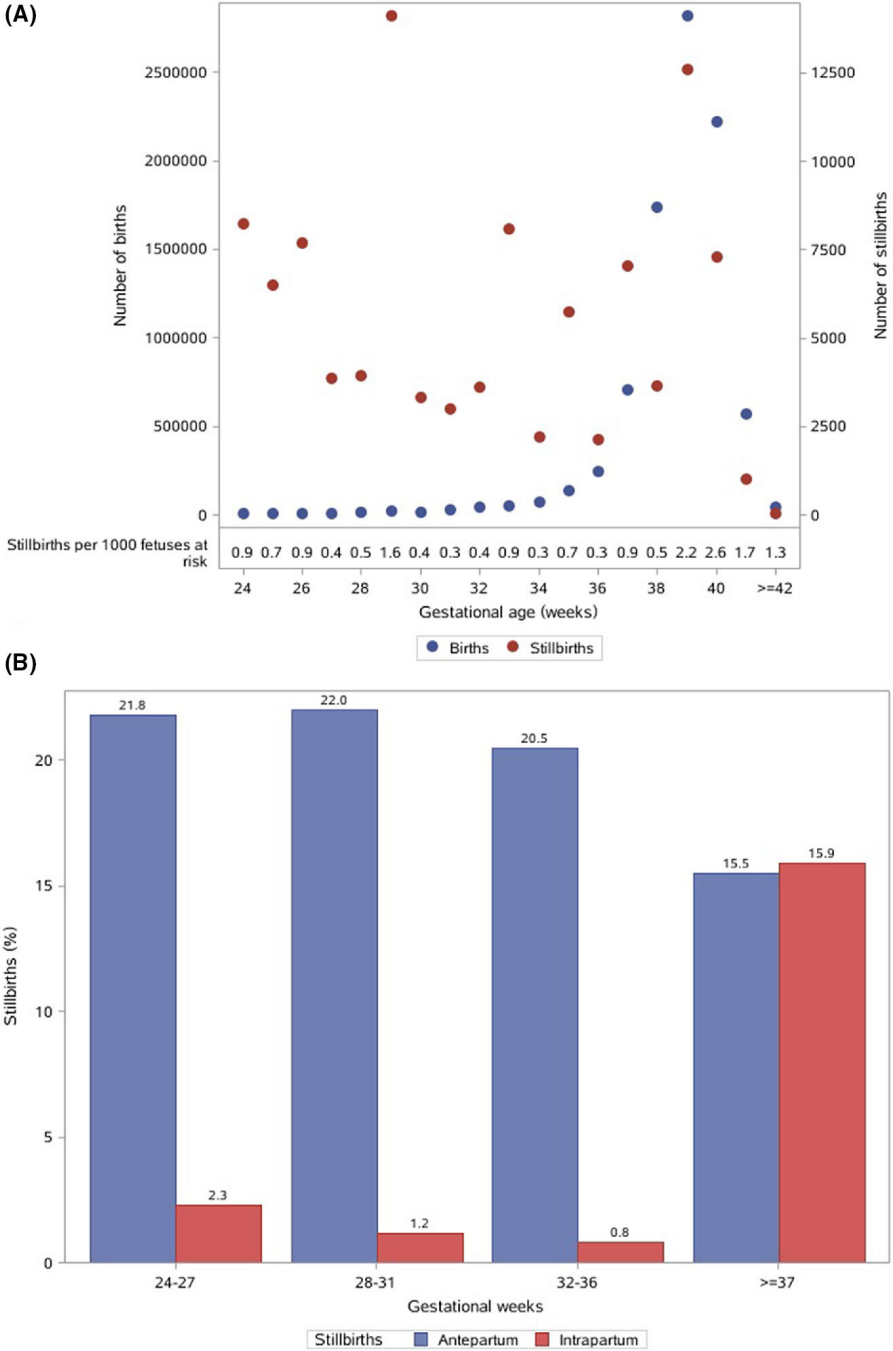
Distribution of births and proportion of stillbirths by week of gestation in China. (A) Distribution of weighted number of births and stillbirths by week of gestation. (B) Weighted proportion of antepartum and intrapartum stillbirths by week of gestation.

A total of 77 879 births were included in the survey. We excluded 2567 births with a gestational age of less than 24 weeks or unknown, and 180 births with unclear fetal outcomes, leaving 75 132 births for the final analysis. There were 949 stillbirths, giving a weighted stillbirth rate of 13.2 per 1000 births (95% CI 7.9–18.5). The distribution of weighted number of births and stillbirths by gestational week is shown in Figure 1A. The weighted proportion of antepartum and intrapartum stillbirth was 78.8% (95% CI 69.2–88.4%) and 21.2% (95% CI 11.6–30.8%), respectively. In all, 24.1% (95% CI 14.5–33.8%) of all stillbirths occurred at 24–27^+6^ weeks of gestation and 31.4% (95% CI 23.4–39.4%) after 37 gestational weeks (Figure 1B). We observed an uneven distribution of stillbirth rates among geographical regions in China, ranging from 9.0 per 1000 births (95% CI 4.3–13.7) in the South to 19.6 per 1000 births (95% CI 10.6–28.5) in the Northwest (Table S1). Low education, pre‐pregnancy obesity, multiparity and previous pregnancy loss were significant risk factors for antepartum stillbirth. Advanced maternal age and previous pregnancy loss were associated with intrapartum stillbirth after controlling for other factors listed in Table S1.

**Figure 2:**
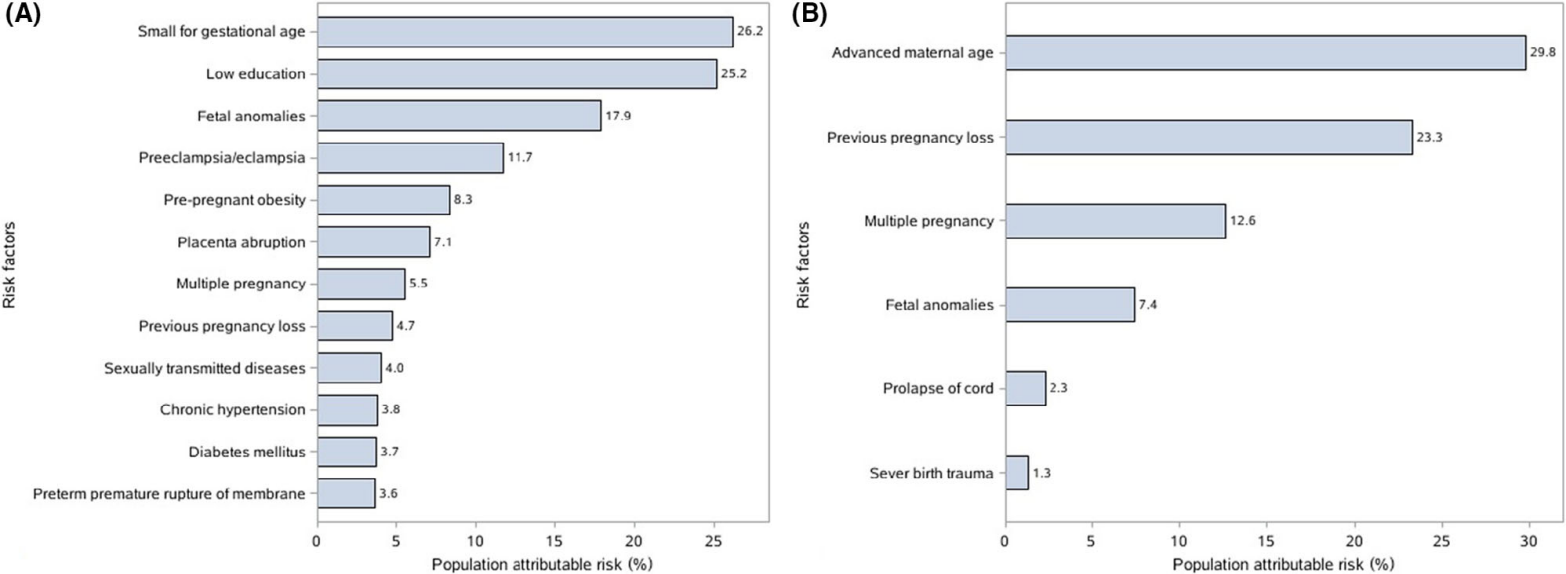
Population attributable risk percentage (PAR%) for antepartum and intrapartum stillbirths according to maternal socio‐demographic characteristics and pregnancy risk factors. (A) PAR% for antepartum stillbirths. (B) PAR% for intrapartum stillbirths.

Table S2 presents the associations between medical conditions and stillbirth, and Figure 2 shows the PAR% of risk factors for antepartum and intrapartum stillbirths at population level. SGA increased antepartum stillbirths by 26.2% (95% CI 25.9–26.5%) compared with pregnancies without SGA. Fetal anomalies were associated with antepartum (adjusted HR 36.5, 95% CI 16.5–80.7) and intrapartum stillbirths (adjusted HR 12.0, 95% CI 1.5–95.2), and increased antepartum and intrapartum stillbirths by 17.9% (95% CI 17.6–18.1%) and 7.4% (95% CI 7.1–7.7%), respectively. Pre‐eclampsia/eclampsia was associated with antepartum stillbirth (adjusted HR 8.27, 95% CI 5.63–12.15) and increased antepartum stillbirths by 11.7% (95% CI 11.4–11.9%), compared with pregnancies without pre‐eclampsia/eclampsia. Multiple pregnancies, compared with singleton pregnancies, increased antepartum and intrapartum stillbirths by 5.5% (95% CI 5.3–5.7%) and 12.6% (95% CI 12.2–13.1%), respectively. Chronic hypertension, placenta abruption, PPROM and sexually transmitted diseases were risk factors for antepartum stillbirth, whereas prolapse of the cord and severe birth trauma were the main risk factors for intrapartum stillbirth. Notably, factors that may be attributable to low education increased antepartum stillbirths by 25.2% (95% CI 24.7–25.6%). The prevalence of advanced maternal age (≥35 years) was 11.0% (95% CI 9.5–12.5%) and increased intrapartum stillbirths by 29.8% (95% CI 29.2–30.5%) compared with a maternal age of less than 35 years.

**Figure 3:**
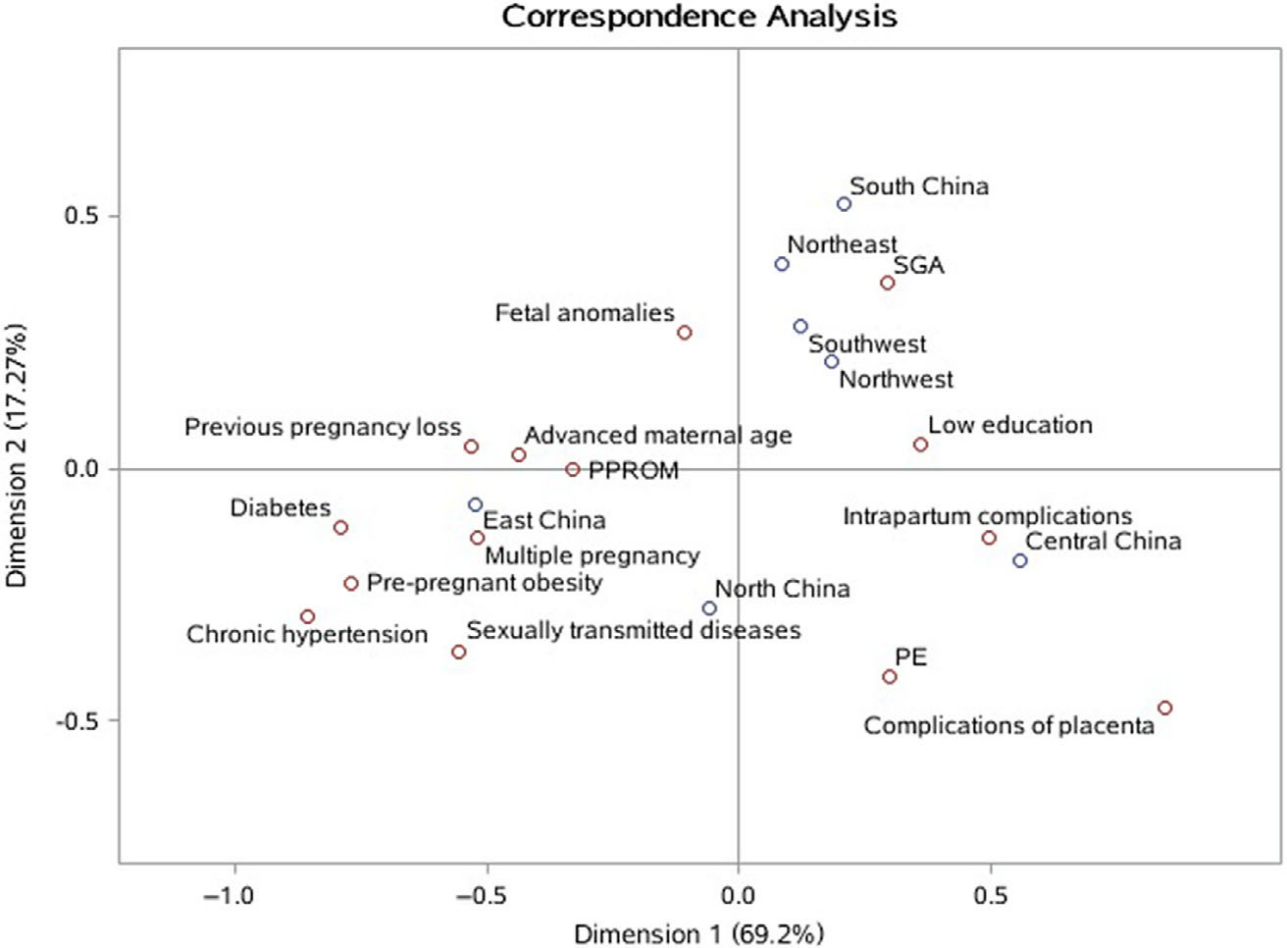
Correspondence analysis of stillbirths between risk factors and geographical regions in China.

We further categorised causes of stillbirths into potentially preventable, fetal and other causes (Figure S1). A weighted proportion of each category in geographical regions in China is shown in Table 1. Overall, 31.4% (95% CI 26.1–36.6%) of all stillbirths were potentially preventable, ranging from 21.0% (95% CI 11.1–30.9%) in the Southwest to 41.5% (95% CI 8.6–74.5%) in the South. Meanwhile, fetal causes and other causes accounted for 16.3% (95% CI 9.7–22.9%) and 52.3% (95% CI 45.8–58.8%) of all stillbirths, respectively. The correspondence analysis examining the association between risk factors and geographical regions in all weighted stillbirths indicates that stillbirths related to advanced maternal age, pre‐pregnant obesity, chronic hypertension, diabetes mellitus, PPROM, sexually transmitted diseases and multiple pregnancy were more frequent in East China than in other regions; low education and SGA were more common in Northwest, Southwest, Northeast and South China than in Central, North and East China; and pre‐eclampsia/eclampsia, placental complications and intrapartum complications were more frequent in Central China than in other regions in China (Figure 3, Table S3).

**Table 1:** Potentially preventable causes for stillbirths by geographical region in China

	Potentially preventable causes		Fetal causes		Other causes	
	Weighted number of stillbirths^a^	% (95% CI)	Weighted number of stillbirths^a^	% (95% CI)	Weighted number of stillbirths^a^	% (95% CI)
East	14 362	32.0 (26.7–37.4)	7872	17.6 (6.0–29.1)	22 613	50.4 (41.4–59.4)
Northeast	1163	29.0 (21.0–36.9)	878	21.9 (16.1–27.6)	1975	49.2 (35.5–62.8)
Northwest	2955	39.2 (32.7–45.6)	691	9.2 (6.2–12.1)	3898	51.7 (47.6–55.7)
Southwest	2309	21.0 (11.1–30.9)	3320	30.2 (17.9–42.5)	5373	48.8 (40.8–56.9)
North	1926	41.3 (24.1–58.6)	677	14.5 (3.3–25.8)	2058	44.1 (34.1–54.2)
Central	9288	27.2 (19.0–35.4)	3715	10.9 (0.0–22.8)	21 169	61.9 (52.5–71.4)
South	5434	41.5 (8.6–74.5)	2309	17.7 (1.3–34.0)	5335	40.8 (11.0–70.6)
Total	37 437	31.4 (26.1–36.6)	19 462	16.3 (9.7–22.9)	62 421	52.3 (45.8–58.8)

Potentially preventable causes include chronic hypertension, diabetes mellitus, hyperthyroidism, hypothyroidism, autoimmune disease, renal disease, Rh incompatibility, gestational hypertension, pre‐eclampsia/eclampsia, gestational diabetes, preterm premature rupture of membrane, SGA, prolapse of cord, fetal heart rate abnormality, shoulder dystocia, sever birth trauma, prolonged labour, sexually transmitted disease, post‐term pregnancy.

Fetal causes include fetal genetic/structural abnormalities.

Other causes include placenta praevia, placenta abruption, multiple pregnancy and other unknown causes.

^a^Adjusted for sampling strategy and clustering of births within hospitals.

## Discussion

### Main findings

Our study including births from 24 weeks’ gestation showed a stillbirth rate of 13.2 per 1000 births (95% CI 7.9–18.5) in China in 2015–2016. The stillbirth rate varied by region, from 9.0 per 1000 births in South China to 19.6 per 1000 births in Northwest China. Of all stillbirths, 24.1% were between 24 and 27^+6^ weeks of gestation and they were mostly antepartum. Antepartum stillbirths accounted for 78.8% (95% CI 69.2–88.4%) of all stillbirths. Nearly one‐third of stillbirths were potentially preventable.

### Strengths and limitations

Our study has several strengths. First, the China Labor and Delivery Survey involved 96 hospitals covering most geographical regions in China. Deliveries at secondary and tertiary hospitals accounted for over 90% of births in China in the last 10 years.^6^ Thus, our results represented the large secondary and tertiary hospital‐based delivery in China as well. Second, our study was one of the first studies considering births from 24 weeks of gestation in China. We used the same data collection form in all hospitals, resulting in more uniformity and reliability for comparisons. The data collection form has been used previously in studies by WHO.^19^ Third, our results highlight the regional diversities in the incidence and risk factors for stillbirth in China. This information is important for local governments to set up even more relevant and, hopefully, more effective intervention strategies for their own regions.

Our study also has some shortcomings. First, detailed information on individual socio‐economic status was not collected in the survey. We could not make an in‐depth assessment on the effect of socio‐economic factors on stillbirth. Second, the definition of ‘potentially preventable causes’ is subjective and open to debate, as more fetal deaths could be avoided due to advances in clinical practice. Finally, our study was a cross‐sectional survey using medical records. Thus, the temporal relation may be distorted in some cases. For example, gestational hypertension often occurs in term and post‐term.^20^ Thus, women who carried the pregnancy to term or later are less likely to experience antepartum stillbirth, which made it look as if gestational hypertension had had a protective effect on stillbirth (Table S2). Likewise, in our population, women who had placenta praevia, prolonged labour, gestational hypertension or gestational diabetes were much more likely to have had a caesarean delivery (results not shown), which made it look like that these conditions had had a protective effect on intrapartum stillbirth. The increased risk of antepartum stillbirth in women with parity 1 compared with parity 0 might be due partly to the high caesarean section rate in the first pregnancy in China, which could increase the risk of unexplained stillbirth in the second.^21^, ^22^, ^23^ Therefore, the interpretation of our findings requires caution.

### Interpretation

Few national or facility‐based stillbirth data are available in China. The previously estimated stillbirth rate for China was 9.0 per 1000 births in 2008 and 9.4 per 1000 births in 2009.^2^, ^24^ Zhu et al.^6^ reported a stillbirth rate of 8.8 per 1000 births (95% CI 8.8–8.9) in 2012–2014. All these estimates were based on births after 28 weeks of gestation.^25^

A recommendation of a 28‐week threshold for international comparison of stillbirths may underestimate the true burden of stillbirths, as a substantial proportion of stillbirths occur between 24 and 28 weeks. Our study found that 24.1% of all stillbirths occurred at 24–27^+6^ gestational weeks. If we exclude these births, China’s stillbirth rate would be 9.6 per 1000 births (95% CI 5.9–13.3) born at 28 weeks of gestation or later, which is quite similar to the previous estimates.

Risk factors for stillbirths vary among high‐, middle‐ and low‐income countries. A 2011 meta‐analysis suggested that advanced maternal age (>35 years), obesity, smoking, SGA, placenta abruption, hypertensive disorders and pre‐existing diabetes were major risk factors for stillbirths in high‐income countries,^26^ whereas poverty, lack of education, low birthweight, diabetes, syphilis, malaria, congenital anomalies, asphyxia, birth trauma and placenta causes were major risk factors for stillbirth in low‐ and middle‐income countries.^27^ Our findings on the variations of risk factors by region are consistent with these patterns. For example, in East China, where the economy is most developed, risk factors such as advanced maternal age, pre‐pregnant obesity, chronic hypertension and diabetes were more common, whereas in the less developed regions in the country, low education, SGA and fetal anomalies were major risk factors, highlighting the importance of site‐specific strategies to tackle the stillbirth problem at the local level.

Small for gestational age was one of the main causes of stillbirth, especially in Northwest, Southwest, Northeast and South China, which is consistent with a previous report.^28^ Maternal complications (e.g. pre‐eclampsia), fetal genetic and structural abnormalities and placental disorders are common aetiologies of SGA.^29^ Ideally, all pregnant women should be screened for risk factors during antenatal visits. Further evaluation should be considered in growth‐restricted fetuses for amniotic fluid assessment and umbilical artery velocimetry, which can improve perinatal outcomes when combined with standard fetal surveillance methods.^30^ In 2016, WHO recommended at least eight antenatal care contacts during pregnancy, including clinical health promotion and prevention and early detection of pregnancy‐related conditions.^31^ This calls for not only having regular prenatal care visits but also improving quality of care to identify growth‐restricted fetus early to prevent stillbirth.

Intrapartum complications were the main causes of stillbirth in Central China. In 2015, Lawn et al.^32^ estimated that 1.3 million babies were stillborn at delivery worldwide, accounting for 49.6% of all stillbirths. In Eastern Asia this figure is estimated at 19.9%. In our study, 21.2% (95% CI 11.6–30.8%) of stillbirths occurred during labour. Our findings were even consistent with those that used different methods (e.g. the International Classification of Diseases‐Perinatal Mortality) to classify antepartum and intrapartum stillbirths.^33^, ^34^ To reduce maternal and neonatal mortality, China has made great efforts to promote hospital‐based birth by strengthening infrastructure, staff training, reducing costs for women in rural areas and establishing referral channels to tertiary hospitals that could handle emergency obstetric care.^35^ Since 2014, 99.6% of all women give birth in hospitals.^36^ Meanwhile, neonatal resuscitation training at county‐level hospitals was initiated in 2004 by the China Ministry of Health. As a result, intrapartum‐related neonatal deaths declined from the leading cause (7.1 per 1000 livebirths in 1995) to the third (1.5 per 1000 livebirths in 2015).^37^ Nonetheless, a recent meta‐analysis suggested that certain areas of China still has a high neonatal mortality rate, with neonatal asphyxia as the leading cause.^38^ Data from seven hospitals in Shanxi Province showed that the quality of emergency obstetric care was often poor and the management of complications was not evidence‐based.^39^ Results from four provinces in China showed that the newborn resuscitation equipment was available for immediate use per WHO recommendations in only 40% of birth asphyxia cases.^40^ Although the overall significant drop in intrapartum stillbirth is commendable, the in‐practice obstetric emergency training or the simulation‐based integrated clinical teamwork training, particularly on the assessment of fetal well‐being during labour according to WHO recommendations,^41^ should be the key to the further reduction in intrapartum stillbirth rate.

## Conclusion

Our study indicated that the prevalence of stillbirth was 13.2 per 1000 births in China in 2015–2016. In addition, nearly one‐third of all stillbirths may be preventable. Strategies based on regional characteristics and risk factors should be considered to further reduce the burden of stillbirths in China.

### Disclosure of interests

The authors declare that they have no conflicts of interests. Completed disclosure of interest forms are available to view online as supporting information.

### Contribution to authorship

Jing Zhu, Hongwei Xia, TD and Jun Zhang conceived the study and provided overall guidance. Hongwei Xia, JG, Xiaodong Ye, BG, ML, LD, LZ, LC, Yun Wang, XW, HL, CC, Yeping Wang, GW, MC, Xiaochang Yang, FL, CF, YR, LY, RZ, Hualin Xu, Jinxiang Zhang, XM, DY, YZ and DW collected data. Jinwen Zhang assisted with data collection and conducted the statistical analysis. Jing Zhu, APB, HQ, TD and Jun Zhang drafted the manuscript and all authors contributed to interpretation of the results and development of the report. All authors reviewed and approved the final version.

### Details of ethics approval

This study was approved by the Ethics Review Board of the Xinhua Hospital Affiliated to the Shanghai Jiao Tong University School of Medicine (XHEC–C–2015–006, 20 February 2015), the WHO Research Ethics Review Committee (HRP Study A65899, 1 May 2015) and participating hospitals.

### Funding

The project was supported in part by Shanghai Municipal Health Commission (GWIV–26.2). The funders did not play any role in the study design, data collection and analysis, decision to publish or preparation of the manuscript.

## Supporting information


**Figure S1.** Flowchart of classification of stillbirths.

**Table S1.** Association between maternal socio‐demographic characteristics and stillbirths in China.
**Table S2.** Associations of medical conditions and stillbirths in China.
**Table S3.** Weighted proportion of stillbirths with the following risk factors by geographical region in China.

Supplementary Material
