# Supplementary figures and images for: Stillbirths in China: a nationwide survey
Source: BJOG. 2020 Sep 2;128(1):67–76. doi: 10.1111/1471-0528.16458 (PMC7754392; doi:10.1111/1471-0528.16458)

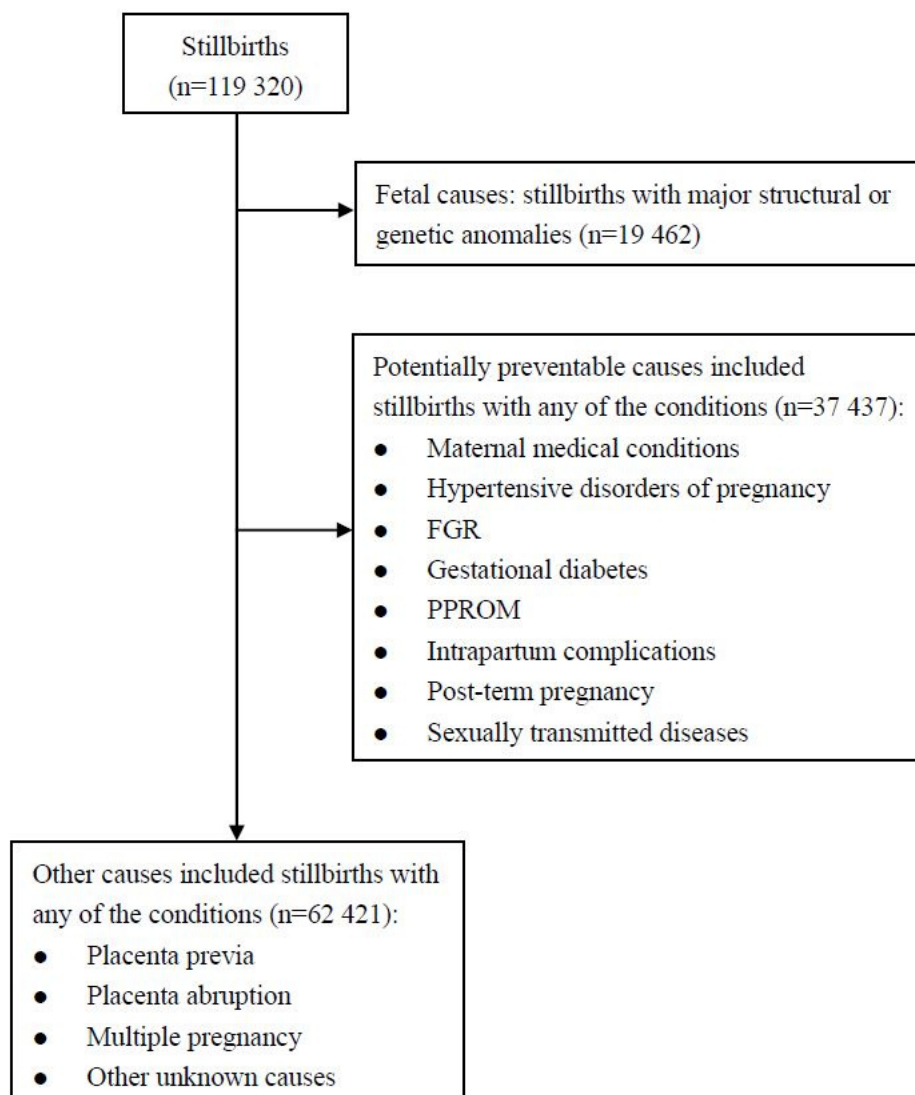

**Figure S1.** Flowchart of classification of stillbirths.

Supplement: Supplementary file 1 — Figure S1. Flowchart of classification of stillbirths. [file BJO-128-67-s001.pdf]

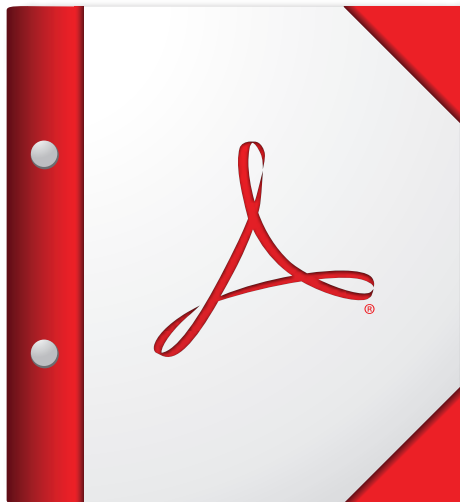

为获得最佳体验，请在 **Acrobat X**、**Adobe Reader X**  
或更高版本中打开此 **PDF** 包。

立即下载 Adobe Reader !

Supplement: Supplementary file 3 — Supplementary Material [file BJO-128-67-s003.pdf]
