# Supplementary material for: Stillbirths in China: a nationwide survey
Source: BJOG. 2020 Sep 2;128(1):67–76. doi: 10.1111/1471-0528.16458 (PMC7754392; doi:10.1111/1471-0528.16458)
Supplement: Supplementary file 2 — Table S1. Association between maternal socio‐demographic characteristics and stillbirths in China. Table S2. Associations of medical conditions and stillbirths in China. Table S3. Weighted proportion of stillbirths with the following risk factors by geographical region in China. [file BJO-128-67-s002.pdf]

Table S1. Association between maternal sociodemographic characteristics and stillbirths in China

	Weighted number of births* (%)	Antepartum stillbirth			Intrapartum stillbirth		
		Weighted number of antepartum stillbirths* (per 1000 births)	Crude HR* (95%CI)	Adjusted HR*† (95%CI)	Weighted number of intrapartum stillbirths* (per 1000 births)	Crude HR* (95%CI)	Adjusted HR*† (95%CI)
Maternal age (years)							
< 25	1 844 072 (20.4)	18 809 (10.2)	1.18 (0.73–1.93)	0.97 (0.55–1.72)	8658 (4.7)	4.52 (1.41–14.50)	5.09 (0.83–31.26)
25-29	4 118 902 (45.6)	36 223 (8.8)	1	1	4280 (1.0)	1	1
30-34	2 073 389 (23.0)	22 459 (10.8)	1.27 (0.89–1.83)	1.27 (0.76–2.13)	2851 (1.4)	1.29 (0.67–2.47)	1.21 (0.64–2.31)
≥ 35	993 327 (11.0)	16 547 (16.7)	1.48 (0.97–2.26)	1.17 (0.79–1.72)	9493 (9.6)	8.24 (2.27–29.89)	7.49 (1.50–37.47)
Race							
Han	8 736 695 (96.8)	90 901 (10.4)	1	1	24 394 (2.8)	1	1
Other	292 995 (3.2)	3137 (10.7)	1.05 (0.49–2.26)	0.87 (0.37–2.06)	888 (3.0)	1.43 (0.50–4.13)	1.53 (0.53–4.36)
Education (years)							
Less than high school (≤9)	3 069 862 (37.8)	47 596 (15.5)	2.50 (1.29–4.84)	2.56 (1.11–5.92)	5431 (1.8)	0.79 (0.33–1.94)	0.85 (0.44–1.67)
High school (10–12)	1 654 160 (20.4)	15 609 (9.4)	1.58 (0.68–3.67)	1.63 (0.61–4.37)	5556 (3.4)	1.59 (0.85–2.98)	1.82 (0.78–4.27)
College and above (>12)	3 388 890 (41.8)	24 499 (7.2)	1	1	10 663 (3.1)	1	1

Social health insurance							
Yes	4 290 039 (61.8)	43 713 (10.2)	1	1	13 363 (3.1)	1	1
No	2 655 824 (38.2)	30 183 (11.4)	1.10 (0.58–2.08)	0.96 (0.49–1.88)	7977 (3.0)	0.55 (0.20–1.50)	0.64 (0.23–1.75)
Pre-pregnant BMI (kg/m^2)							
< 18.5	779 866 (13.1)	3602 (4.6)	0.83 (0.46–1.51)	1.03 (0.60–1.76)	686 (0.9)	0.25 (0.06–1.02)	0.29 (0.07–1.18)
18.5–23.9	3 946 038 (66.2)	24 587 (6.2)	1	1	16 372 (4.1)	1	1
24.0–27.9	974 487 (16.3)	5013 (5.1)	0.98 (0.51–1.87)	0.90 (0.43–1.88)	1394 (1.4)	0.44 (0.11–1.69)	0.38 (0.07–2.16)
≥ 28.0	261 568 (4.4)	4677 (17.9)	3.45 (1.13–10.56)	2.84 (1.12–7.22)	1756 (6.7)	2.18 (0.37–12.93)	2.49 (0.37–16.53)
Parity							
0	4 325 317 (48.1)	31 085 (7.2)	1	1	10 186 (2.4)	1	1
1	3 589 178 (39.9)	52 542 (14.6)	2.45 (1.29–4.68)	1.90 (1.19–3.03)	8919 (2.5)	1.12 (0.60–2.09)	1.24 (0.71–2.18)
≥ 2	1 078 357 (12.0)	8896 (8.2)	1.38 (0.67–2.83)	0.84 (0.32–2.17)	5788 (5.4)	1.15 (0.30–4.39)	1.28 (0.49–3.33)
ART	590 341 (6.5)	7000 (11.9)	0.61 (0.30–1.21)	0.51 (0.23–1.10)	3480 (5.9)	2.80 (0.65–12.04)	0.74 (0.22–2.54)
Pregnancy history							
Previous pregnancy loss	669 269 (7.4)	11 023 (16.5)	1.70 (1.00–2.90)	1.60 (1.01–2.68)	7322 (10.9)	3.01 (0.83–10.85)	3.58 (1.00–12.88)
Previous stillbirth	103 315 (1.1)	2183 (21.1)	2.07 (0.90–4.76)	1.61 (0.67–3.92)	212 (2.1)	0.85 (0.17–4.35)	0.93 (0.18–4.95)
Previous preterm birth	129 726 (1.4)	1654 (12.8)	1.43 (0.58–3.50)	0.94 (0.35–2.51)	162 (1.2)	0.38 (0.07–1.99)	0.21 (0.04–1.28)
Hospital level							

Level 2	4 843 524 (53.6)	34 044 (7.0)	1	1	8365 (1.7)	1	1
Level 3	4 186 167 (46.4)	59 994 (14.3)	1.94 (0.78–4.84)	3.25 (1.35–7.83)	16 917 (4.0)	1.98 (0.72–5.42)	2.26 (0.80–6.44)
Region							
East	3 541 062 (39.2)	31 955 (9.0)	1	1	12 892 (3.6)	1	1
Northeast	429 349 (4.8)	3133 (7.3)	0.89 (0.13–6.12)	0.84 (0.10–6.98)	883 (2.1)	0.68 (0.15–3.20)	0.64 (0.12–3.44)
Northwest	385 581 (4.3)	6691 (17.4)	1.90 (0.73–4.93)	2.23 (0.92–5.44)	853 (2.2)	0.62 (0.21–1.82)	0.76 (0.30–1.93)
Southwest	784 908 (8.7)	8722 (11.1)	1.24 (0.43–3.59)	0.90 (0.24–3.46)	2280 (2.9)	1.08 (0.34–3.46)	0.99 (0.27–3.67)
North	399 044 (4.4)	3399 (8.5)	1.09 (0.38–3.14)	1.07 (0.32–3.59)	1262 (3.2)	1.24 (0.31–5.00)	1.16 (0.33–4.07)
Central	2 032 256 (22.5)	30 517 (15.0)	1.67 (0.53–5.27)	1.24 (0.33–4.76)	3655 (1.8)	0.53 (0.13–2.25)	0.38 (0.07–1.99)
South	1 457 490 (16.1)	9621 (6.6)	0.79 (0.26–2.40)	0.76 (0.25–2.36)	3457 (2.4)	0.93 (0.34–3.46)	0.27 (0.08–0.94)

BMI, body mass index; ART, assisted reproductive technology; HR, hazard ratio; CI, confidence interval.

*Adjusted for sampling distribution.

†Adjusted for maternal age, race, insurance, education, parity, pre-pregnant BMI, previous loss, previous stillbirth, previous preterm birth, hospital location and hospital levels.

Table S2. Associations of medical conditions and stillbirths in China

	Weighted number of births* (%)	Antepartum stillbirth			Intrapartum stillbirth		
		Weighted number of antepartum stillbirths* (per 1000 births)	Crude HR* (95%CI)	Adjusted HR*† (95%CI)	Weighted number of intrapartum stillbirths* (per 1000 births)	Crude HR* (95%CI)	Adjusted HR*† (95%CI)
Maternal medical conditions							
Chronic hypertension	40 643 (0.5)	4017 (98.8)	13.5 (4.3–42.8)	9.89 (3.22–30.4)	170 (4.2)	3.42 (0.57–20.70)	2.77 (0.44–17.31)
Diabetes mellitus	86 233 (1.0)	4329 (50.2)	1.19 (0.44–3.18)	0.88 (0.22–3.49)	282 (3.3)	2.27 (0.42–12.25)	1.06 (0.21–5.32)
Other maternal complications	248 111 (2.7)	1584 (6.4)	0.64 (0.27–1.53)	0.64 (0.23–1.79)	81 (0.3)	0.15 (0.03–0.80)	0.18 (0.03–1.02)
Pregnancy complications							
Gestational hypertension	145 742 (1.6)	584 (4.0)	0.44 (0.18–1.06)	0.25 (0.09–0.65)	64 (0.4)	0.21 (0.04–0.99)	0.11 (0.01–0.87)
Preeclampsia/eclampsia	215 380 (2.4)	12 940 (60.1)	8.98 (5.46–14.76)	8.27 (5.63–12.15)	901 (4.2)	2.91 (0.96–8.84)	2.68 (0.80–8.94)
Gestational diabetes	938 344 (10.4)	10 003 (10.7)	1.15 (0.62–2.14)	1.29 (0.68–2.46)	569 (0.6)	0.28 (0.06–1.25)	0.25 (0.04–1.47)
Placenta previa	121 322 (1.3)	2543 (21.0)	2.52 (1.20–5.33)	2.13 (0.82–5.50)	76 (0.6)	0.52 (0.11–2.61)	0.14 (0.01–1.40)
Placenta abruption	61 824 (0.7)	7321 (118.4)	12.7 (2.6–62.7)	14.3 (3.1–66.7)	357 (5.8)	3.84 (0.87–16.95)	4.95 (1.13–21.66)
PPROM	188 806 (2.1)	5305 (28.1)	4.45 (1.86–	4.58 (1.92–	756 (4.0)	11.1 (2.6–48.1)	9.73 (1.70–55.82)

			10.67)	10.90)			
SGA	247 548 (2.8)	23 609 (95.4)	16.9 (11.6–24.6)	16.0 (10.3–24.9)	1044 (4.2)	2.26 (0.79–6.51)	2.61 (0.67–10.20)
Intrapartum complications							
Uterine rupture	16 306 (0.2)	0	NA	NA	0	NA	NA
FHR abnormality	577 878 (6.4)	8866 (15.3)	1.65 (0.62–4.40)	1.81 (0.66–4.93)	2441 (4.2)	1.73 (0.47–6.42)	2.31 (0.47–11.48)
Prolonged labor	322 966 (3.6)	0	NA	NA	175 (0.5)	0.16 (0.04–0.75)	0.08 (0.02–0.41)
Prolapse of cord	8253 (0.1)	0	NA	NA	600 (72.7)	54.5 (15.4–192.4)	74.6 (22.6–246.0)
Shoulder dystocia	25 829 (0.3)	0	NA	NA	285 (11.0)	4.32 (1.28–14.63)	1.86 (0.22–15.49)
Sever birth trauma	13 091 (0.1)	0	NA	NA	358 (27.3)	6.94 (1.42–33.96)	15.3 (3.8–61.9)
Post-term pregnancy	41 294 (0.5)	52 (1.3)	0.0	0.0	0	NA	NA
Sexually transmitted diseases	69 498 (0.8)	4439 (63.9)	7.40 (1.84–29.77)	7.62 (1.99–29.20)	0	NA	NA
Multiple pregnancy	166 016 (1.8)	6792 (40.9)	3.04 (1.12–8.20)	2.54 (0.76–8.47)	3596 (21.7)	2.69 (0.99–7.29)	2.07 (0.63–6.85)
Fetal genetic/structural abnormalities	67 901 (0.8)	17 412 (256.4)	38.6 (16.1–92.7)	36.5 (16.5–80.7)	2049 (30.2)	12.7 (3.1–52.1)	12.0 (1.5–95.2)

PPROM, preterm premature rupture of membrane; SGA, small for gestational age; FHR, fetal heart rate; HR, hazard ratio; CI, confidence interval; NA, not applicable.

*Adjusted for sampling distribution.

†Adjusted for maternal age, race, insurance, education, parity, pre-pregnant BMI, previous loss, previous stillbirth, previous preterm birth, hospital location and hospital levels.

Other maternal complications: including hyperthyroidism, hypothyroidism, autoimmune disease, renal disease and RH incompatibility.

Table S3. Weighted proportion of stillbirths with the following risk factors by geographical regions in China

Risk factors	East	Northeast	Northwest	Southwest	North	Central	South
	**N (%) ***	**N (%) ***	**N (%) ***	**N (%) ***	**N (%) ***	**N (%) ***	**N (%) ***
Advanced maternal age (≥ 35 years)	14 656 (37.8)	1305 (33.4)	571 (7.6)	1986 (18.5)	388 (8.3)	1665 (8.2)	1803 (14.8)
Low education (less than high school)	10 787 (27.8)	1284 (32.9)	3070 (40.7)	5247 (48.9)	1048 (22.5)	14 022 (69.3)	6347 (52.2)
Pre-pregnant obesity (BMI ≥ 28.0 kg/m^2)	4578 (11.8)	71 (1.8)	254 (3.4)	233 (2.2)	646 (13.9)	138 (0.7)	164 (1.3)
Previous pregnancy loss	10 835 (27.9)	641 (16.4)	807 (10.7)	1238 (11.5)	422 (9.1)	1745 (8.6)	1209 (9.9)
Chronic hypertension	3110 (8.0)	0 (0)	0 (0)	237 (2.2)	458 (9.8)	13 (0.1)	62 (0.5)
Diabetes mellitus	3160 (8.1)	0 (0)	0 (0)	138 (1.3)	49 (1.0)	39 (0.2)	334 (2.7)
Preeclampsia/eclampsia	3449 (8.9)	356 (9.1)	553 (7.3)	631 (5.9)	1204 (25.9)	4591 (22.7)	217 (1.8)
Complications of placenta	581 (1.5)	214 (5.5)	290 (3.8)	510 (4.7)	229 (4.9)	4360 (21.6)	257 (2.1)
PPROM	3259 (8.4)	71 (1.8)	773 (10.2)	147 (1.4)	33 (0.7)	533 (2.6)	427 (3.5)
SGA	5064 (13.6)	1519 (39.1)	2149 (30.7)	2451 (28.3)	857 (23.0)	5258 (31.6)	4443 (37.6)
Intrapartum complications	1839 (4.7)	285 (7.3)	817 (10.8)	1524 (14.2)	405 (8.7)	4198 (20.8)	642 (5.3)
Sexually transmitted disease	3062 (7.9)	0 (0)	0 (0)	125 (1.2)	34 (0.7)	786 (3.9)	31 (0.3)
Multiple pregnancy	6437 (16.6)	0 (0)	835 (11.1)	569 (5.3)	320 (6.9)	723 (3.6)	341 (2.8)
Fetal genetic/structural abnormalities	5405 (13.9)	878 (22.5)	691 (9.2)	3320 (30.9)	677 (14.5)	2572 (12.7)	2299 (18.9)

PPROM, preterm premature rupture of membrane; SGA, small for gestational age; .

*Adjusted for sampling strategy and clustering of births within hospitals.

Complications of placenta include placenta previa and placenta abruption.

Intrapartum complications include prolapse of cord, fetal heart rate abnormality, shoulder dystocia and sever birth trauma.
